# Characteristics of Low Reynolds Number Shear-Free Turbulence at an Impermeable Base

**DOI:** 10.1155/2014/683537

**Published:** 2014-08-27

**Authors:** W. H. M. Wan Mohtar, A. ElShafie

**Affiliations:** Department of Civil & Structural Engineering, Faculty of Engineering and Built Environment, Universiti Kebangsaan Malaysia, 43600 Bangi, Malaysia

## Abstract

Shear-free turbulence generated from an oscillating grid in a water tank impinging on an impermeable surface at varying Reynolds number 74 ≤ *Re*_*l*_ ≤ 570 was studied experimentally, where the Reynolds number is defined based on the root-mean-square (r.m.s) horizontal velocity and the integral length scale. A particular focus was paid to the turbulence characteristics for low *Re*_*l*_ < 150 to investigate the minimum limit of *Re*_*l*_ obeying the profiles of rapid distortion theory. The measurements taken at near base included the r.m.s turbulent velocities, evolution of isotropy, integral length scales, and energy spectra. Statistical analysis of the velocity data showed that the anisotropic turbulence structure follows the theory for flows with *Re*_*l*_ ≥ 117. At low *Re*_*l*_ < 117, however, the turbulence profile deviated from the prediction where no amplification of horizontal velocity components was observed and the vertical velocity components were seen to be constant towards the tank base. Both velocity components sharply decreased towards zero at a distance of ≈1/3 of the integral length scale above the base due to viscous damping. The lower limit where *Re*_*l*_ obeys the standard profile was found to be within the range 114 ≤ *Re*_*l*_ ≤ 116.

## 1. Introduction

The interaction of turbulence with an impermeable surface is important in various geophysical phenomena, notably in the turbulence-sediment interaction including incipient sediment motion and sediment entrainment. An in-depth understanding of the physics of near-wall turbulence is essential to better understand the phenomena. Considerable research has been conducted to investigate the characteristics of turbulence at near impermeable surface, where the turbulence is generated using an oscillating grid in a water tank [1–3]. The grid-generated flow (without the presence of an impermeable boundary) is a statistically stationary, zero-mean turbulence that is horizontally homogeneous at depths beyond 2 to 3 mesh widths (*M*) below the grid's mid position. At this distance, the decay of the time-averaged r.m.s velocity components (*u*, *v*, *w*), with depth *z* below the grid's mid position, can be represented by the expression

(1a) $$\begin{array}{c} u=v=C_{1}f_{g}S^{3/2}M^{1/2}z^{-1}, \end{array}$$

(1b) $$\begin{array}{c} w=C_{2}f_{g}S^{3/2}M^{1/2}z^{-1}, \end{array}$$

where *f*_*g*_ is the frequency of oscillation, *S* is the stroke (twice the amplitude), and *C*_1_ and *C*_2_ are the horizontal and vertical constants, usually taken as 0.25 and 0.27, respectively [4]. The derivation of (1a) and (1b) requires the assumption that the horizontal integral length scale of the turbulence *l* satisfies

(2) $$\begin{array}{c} l=\beta z, \end{array}$$

where *β* is constant and usually takes the value 0.1 [5].

Thus, the level of turbulence intensity can be described in terms of Reynolds number *Re*_*l*_ = 2*ul*/*ν*, where *ν* is the water kinematic viscosity. Note that within the quasi-isotropic, quasihomogeneous region at *z* ≳ 2.5*M*, due to the decreasing *u* and increasing *l*, respectively, the Reynolds number *Re*_*l*_ remains constant.

When an initially quasi-isotropic turbulence field is made to interact with a solid boundary, the flow at the near impermeable surface region responds in two ways. First, the decreasing normal velocity component due to the kinematic boundary condition and the intercomponent energy transfer results in an amplification of the tangential velocity components. This wall blocking effect occurs within the boundary layer known as a blockage layer, where the thickness depends on the integral length scale *l*. Second, the no slip condition cancels all velocity components within the thinner viscous layer adjacent to the solid wall. These profile changes has been theoretically described by Hunt and Graham [6] who utilised the methods of rapid distortion theory. Their model describes the inviscid process of an instantaneous response of a turbulent shear-free boundary layer when an impermeable surface is inserted. The theory predicts that the tangential turbulent intensities increase at the expense of the normal components at near surface. At high *Re*_*l*_, this standard profile of the fluctuating field in the bounded shear-free flow has been experimentally and numerically validated [3, 7, 8].

Utilising grid-generated turbulence has been proven to be a successful alternative to examine the sole effect of turbulent fluctuations in various geophysical phenomena such as incipient sediment motion, turbulence-particle effect in suspension, and sedimentation of particles in a water column [9–11]. The statistically stationary grid-generated flow is easy to measure and offers a readily repeatable flow condition, allowing the method to be exploited. Investigating the effect of turbulence on such phenomena, the level of turbulence intensity needed spans from low to high *Re*_*l*_. At low *Re*_*l*_, however, Perot and Moin [12], in their simulation work, conjectured that there is no amplification of the tangential components. In such flow, the properties of statistical turbulence at near impermeable surface deviate from the predicted profile. As such, any geophysical phenomena investigated under (very) low *Re*_*l*_ require a different interpretation of the flow behaviour. Thus, the minimum *Re*_*l*_ needs to be established when turbulent flow used to investigate the geophysical phenomena is generated by an oscillating grid.

Experimental analyses by McDougall [1] and Fernando et al. [13] suggest that the *u* amplification within the near solid region does not occur for flow within the range 40 < *Re*_*l*_ < 150, and this is served as an initial basis. This range, however, is a preconception and cannot as yet be asserted with confidence. The experiments reported here address this preconception by investigating the turbulence properties including the evolution of r.m.s fluid velocity, integral length scales, isotropy, and frequency spectrum; in particular the impermeable surface induced inhomogeneity for flow *Re*_*l*_ < 150.

This paper is set out as follows. In Section 2 of this paper the experimental method is described, and the statistical properties of base-induced turbulence at high *Re*_*l*_ (to verify the experimental setup adopted) are also presented.  Section 3 is devoted to presenting turbulence measurements at low *Re*_*l*_ and comparing them with both existing theoretical equation and turbulence profiles at high *Re*_*l*_. A summary of the results is given in Section 4.

## 2. Experiments

### 2.1. Apparatus

**Figure 1:**
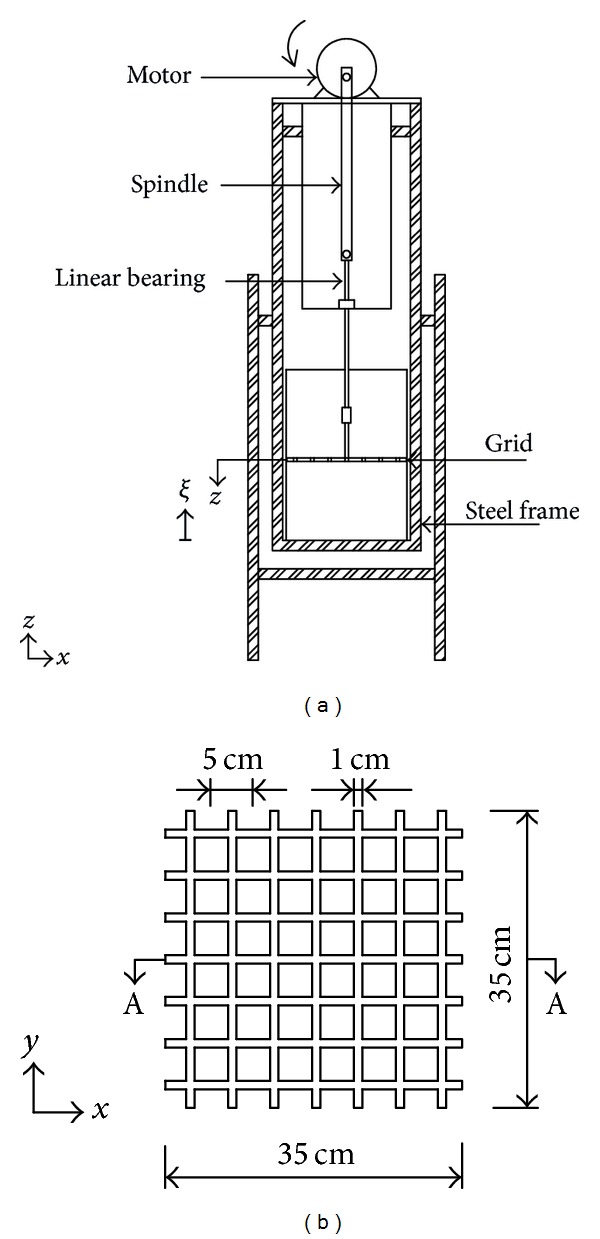
Sketches showing (a) the experimental setup and (b) a (magnified) plan view of the uniform grid. A sediment bed is placed at the bottom of the tank. The symbol *ξ* denotes the distance from sediment bed, where *ξ* = 0 is the tank base.

A set of experimental data was performed to investigate the turbulence profile within the bulk flow, focusing on the region at near base. A sketch of the apparatus is shown in Figure 1. The experiments were performed in an acrylic tank, with cross-sectional dimensions of 35.4 × 35.4 cm^2^ and a height of 50 cm. The tank was fixed within a rigid inner steel frame and filled with water to a depth of 40 cm. The oscillating-grid mechanism, consisting of a linear bearing, was positioned vertically through the central axis of the tank and connected to a rotating spindle. A motor was used to rotate the spindle and thus continuously oscillate the grid. The plan view of the square (*d* = 1 cm) uniform grid shown in Figure 1(b) consisted of a 7 × 7 array of aluminium bars with a length of 35 cm. The uniform mesh size *M* was 5 cm giving the grid a solidity of 36%, within the acceptable solidity limit [4, 14]. The end condition of the grid was designed to ensure that the wall acted as a plane of reflection-symmetry, which has been shown to lessen the Reynolds-stress gradients within the fluid, thereby inhibiting the presence of undesirable secondary circulations (see Fernando and de Silva [14] for a detailed discussion). The opening between the (side) wall and the grid is approximately 2 mm, which was too small to cause any significant secondary circulation [15]. For consistency, each experiment was performed with the stroke fixed at *S* = 8 cm with the grid lowered down so that the distance between the midplane of grid and the base tank is *z* = 3.2*M* = 16 cm. As the stroke was fixed throughout, the turbulence intensity was varied by adjusting the frequency of oscillation within the range 0.8 < *f* ≤ 3.1 Hz.

For each experiment, the velocity measurements (between the grid and the tank base) were obtained using two-dimensional planar particle image velocimetry (PIV), applied in the vertical (*x*, *z*)-plane through the centre of the tank (*y* = 0). The reflective, neutrally buoyant tracer particles were illuminated using a narrow, vertical light sheet (with a thickness of 2 mm) that was directed through the midplane of the tank (Section A-A as shown in Figure 1(b)). The motion of the tracer particles was recorded using a high-speed Dantec NanoSense MkIII digital camera (with 1280 × 1024 pixel resolution) and the frame rate was set at 200 Hz. The camera was positioned so as to have a horizontal view through the tank sidewall and be normal to the vertical light sheet. The camera was connected to a computer, which was used to acquire and store the images. In each case, the instantaneous velocity fields $u=(\tilde{u},\tilde{w})$ were calculated from pairs of images with a cross-correlation algorithm using the Digiflow PIV software [16]. The interrogation area was set at 20 × 20 pixels (equivalent to 3.8 × 3.8 mm^2^), with a 50% overlap between the analysis spots. For each experimental setup, the velocity fields were acquired for a period *T* = 120 seconds, which provided the spatial and the temporal data of the velocity motions in the bulk fluid. Postprocessing of the images showed that mean and r.m.s velocity components were essentially converged after time averaging over a period of at least 80 seconds. The measurement of velocity using PIV allowed a planar flow distribution to be obtained, from the bottom of the grid (i.e., *z* = *S*/2 + *d*/2) to the tank base, giving a physical region of approximately 160 × 200 mm^2^. Thus, the horizontal mean *U*(*x*, *z*) and r.m.s *u*(*x*, *z*) velocity components were obtained by temporally averaging over the acquired period. Note that similar procedure was applied for the mean and r.m.s vertical velocity components.

### 2.2. The Statistical Properties of Base-Induced Inhomogeneous Turbulence

The inhomogeneity behaviour within the blockage layer has been studied extensively using experimental apparatus similar to those described above and by numerical modelling [1, 3, 13]. This section presents the results of a series of experiments, that is, flow with high *Re*_*l*_, to investigate and compare the basic statistical properties of the turbulence generated within the previous experimental studies and the theoretical prediction of Hunt and Graham [6]. However, prior to further analysis, it is practical to examine the significance of the mean flow occurrence in the generated grid turbulence.

**Figure 2:**
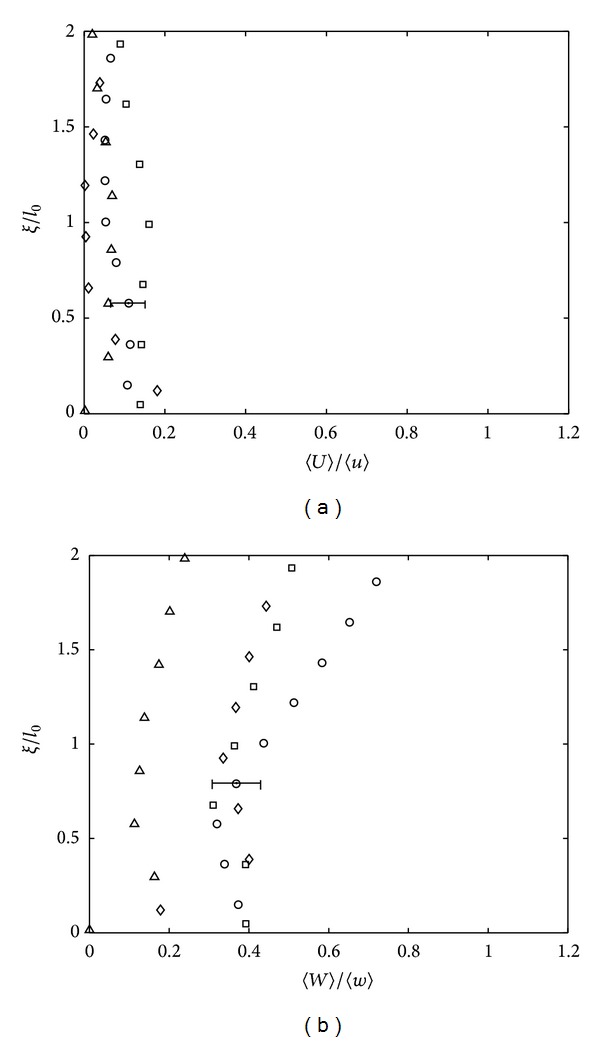
Plots (a) and (b) show the turbulence intensities 〈*U*〉/〈*u*〉 and 〈*W*〉/〈*w*〉 against dimensionless height *ξ*/*l*_0_. The four experiments shown correspond to *Re*_*l*_ = 570 (∘), *Re*_*l*_ = 325 (□), *Re*_*l*_ = 209 (▵), and *Re*_*l*_ = 117 (*⋄*). To avoid saturation, a single error bar has been included in each plot which is representative of the variability observed in all of the data shown.

The weak, secondary mean flow is an inherent characteristic of an oscillating-grid turbulence experiment [17]. Therefore, although several design restrictions have been met (i.e., grid solidity less than 40%, oscillation frequency less than 6 Hz, and very small end openings), it is important to assess the turbulence intensities, in particular at near-base to ensure that the effect of the mean flow is insignificant. Using the operator 〈·〉 to denote spatial averaging, the r.m.s horizontal velocity components were spatially averaged across the horizontal direction *x*, defined as

(3) $$\begin{array}{c} \langle u\left(z\right)\rangle =\frac{1}{2L}\overset{L}{\underset{-L}{\int }}u\left(x,z\right)dx, \end{array}$$

where *L* is the horizontal width from the central midplane of the tank, with typical value of 2*L* = 200 mm.  Figure 2 shows that the presence of base only results in a weak secondary flow, evident by the nonzero values of the horizontal (〈*U*〉) and vertical (〈*W*〉) mean velocity components in the near-base region. However, the ratios 〈*U*〉/〈*u*〉 and 〈*W*〉/〈*w*〉 are typically between 0.1 and 0.4, indicating that the fluctuating components are always dominant compared to the mean components, and so the secondary flow has a minimal impact on the analysis.

**Figure 3:**
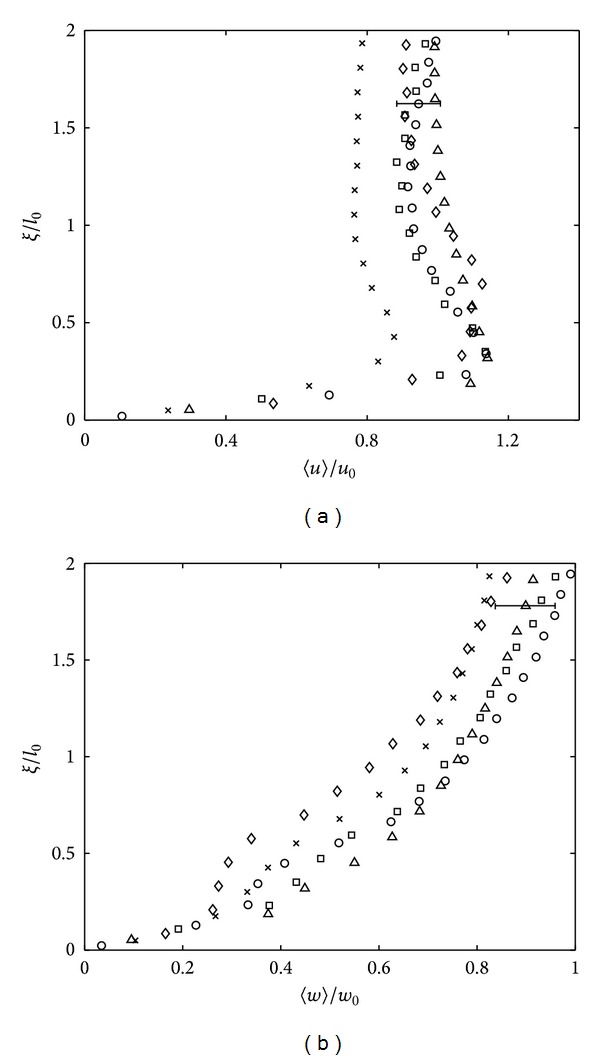
Plots showing measurements of (a) 〈*u*〉/*u*_0_ and (b) 〈*w*〉/*w*_0_ against dimensionless *ξ*/*l*_0_, distance above the tank base. The experiments shown correspond to *Re*_*l*_ = 570 (∘), *Re*_*l*_ = 474 (□), *Re*_*l*_ = 325 (▵), *Re*_*l*_ = 209 (*⋄*), and *Re*_*l*_ = 117 (×). To avoid saturation, a single error bar has been included in each plot which is representative of the variability observed in all of the data shown.

Figure 3 plots both dimensionless r.m.s horizontal 〈*u*〉 and vertical 〈*w*〉 velocity against *ξ*, which henceforth denotes height above and normal to the base surface. The plots show a typical set of velocity profiles within the blockage layer, that is, at *ξ*/*l*_0_ = 0–2. The data is made dimensionless with *u*_0_, *w*_0_, and *l*_0_, which are the values of 〈*u*〉, 〈*w*〉, and *l* measured at depth *z* = 2.5*M* (i.e., *ξ* = 0.9*M*), respectively. The measured values of *u*_0_, *w*_0_, and *l*_0_ for each set of experiment, which were used in the plots presented in this paper, are presented in Table 1. These data also explicitly show, as expected, that both *u*_0_ and *w*_0_ decreased as *Re*_*l*_ decreased.

Figure 3(a) suggests that within the blockage layer the 〈*u*〉 component experiences an amplification between 0.3 ≤ *ξ* ≤ 1, attaining a peak value at *ξ*/*l*_0_ ≈ 0.3. The amplification of the horizontal component is the result of an intercomponent energy transfer produced when the turbulent eddies propagate towards and impinge on the tank base [12]. The energy obtained is transferred from normal velocity components where, as shown in Figure 3(b), the 〈*w*〉 components are progressively damped as the distance to the base decreases *ξ* → 0. The strong self-damping of the normal turbulent intensity within the blockage layer is due to the kinematic boundary condition [12]. The impingement of large-scale turbulent eddies eddy stretches the tangential vorticity components, amplifying the small-scale horizontal turbulence, increasing the horizontal velocity components, denoted here as 〈*u*_*p*_〉 [18]. The distance *ξ* ≈ 0.3 where the near base peak 〈*u*_*p*_〉 is observed lies within the range obtained by previous studies 0.2 ⩽ *ξ* ⩽ 0.4 [7, 19]. At this distance, the maximum amplification observed is about 20% higher than *u*_0_ when *Re*_*l*_ > 120, although for *Re*_*l*_ < 120, the 〈*u*〉 amplified is 〈*u*_*p*_〉/*u*_0_ < 1. This result is in line with Hunt and Graham [6] in the fact that the intensity of amplification depends on the strength of incoming turbulence, where increasing the Reynolds number by a factor of 10 would result in an amplification of 1.3*u*_0_. For all the data, the peak 〈*u*〉 observed was within the range of 0.8*u*_0_ to 1.2*u*_0_.

The r.m.s vertical velocity *w* at near impermeable surface was seen to monotonically decrease to zero at the boundary. Hunt and Carlotti [18] reported that although there might be an amplification of *w* component, it is not significant due to the wall blocking effect. It is clear from Figure 3 that the profiles for both 〈*u*〉 and 〈*w*〉 components agree reasonably well with the theoretical prediction, although the magnitude for the velocity components was much lower as *Re*_*l*_ decreased. Note that the measured 〈*u*〉 and 〈*w*〉 were lower up to a factor of 2 from the predicted values when *Re*_*l*_ = 117. Note that for the range of *Re*_*l*_ > 117 used in this experiment, consistently similar trends to the data presented in Figures 3(a) and 3(b) were observed.

**Figure 4:**
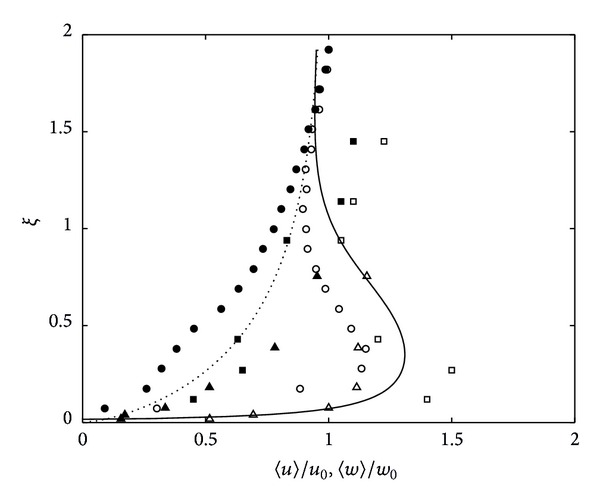
Typical r.m.s velocity profiles at near tank base where data shown is generated from flow *Re*_*l*_ = 570 (∘), plotted together with data extracted from Brumley (▵) and Hannoun (□). The predicted lines derived by Hunt and Graham [6] are also shown as solid line and dashed line for *u* and *w* velocity components, respectively. Open bullets and filled bullets represent the dimensionless r.m.s horizontal and vertical components, respectively.

The same data of *Re*_*l*_ = 570 in Figure 3 was also compared with the results from previous experimental work and the theoretical prediction of Hunt and Graham [6], as shown in Figure 4, to assess the similarity profiles obtained with the previous work. The experimental data from previous work was obtained from Brumley and Jirka [19] and Fernando et al. [13], who analysed the interaction of oscillating grid turbulence with a free-surface and a solid plate, respectively. Although the dynamic boundary condition for a free surface (i.e., the work of Brumley and Jirka [19]) is different (where only *w* = 0), it appears that the anisotropy of the fields is insensitive to the precise nature of the dynamic boundary condition across the blockage layer [3]. Thus, a comparison of data with the velocity profile obtained by Brumley and Jirka [19] is allowed. Figure 4 shows that the previous data and measured r.m.s horizontal velocity components produced similar amplification profiles. From 〈*u*〉/*u*_0_ = 1 at *ξ* = 1, the 〈*u*〉/*u*_0_ amplified in the blockage layer reaching a maximum peak at *ξ* = 0.2–0.4. On the other hand, the 〈*w*〉/*w*_0_ experienced a monotonous decrease towards the surface.

Next, the measurement of the integral length scale, for both horizontal *l* and vertical length *l*_*z*_ scales, was computed. Note that the symbol for the horizontal integral length scale is *l*, to be in line with other research and, for the vertical integral length scale, the subscript *z* was added. These measures give both the quantitative turbulent eddy sizes available and how the base modifies the turbulent structure. The horizontal integral length scales were calculated by computing the autocorrelation function based on the r.m.s horizontal (*u*) velocity components, expressed as

(4) $$\begin{array}{c} R_{uu}\left(r,x\right)=\frac{\bar{u(x)u(x+r)}}{\bar{u^{2}}}, \end{array}$$

and by obtaining the integral of *R*_*uu*_(*r*) to the first zero crossing. Similar procedure was applied to obtain the vertical integral length scale *l*_*z*_ using the autocorrelation function based on the r.m.s vertical (*w*) velocity components.

**Figure 5:**
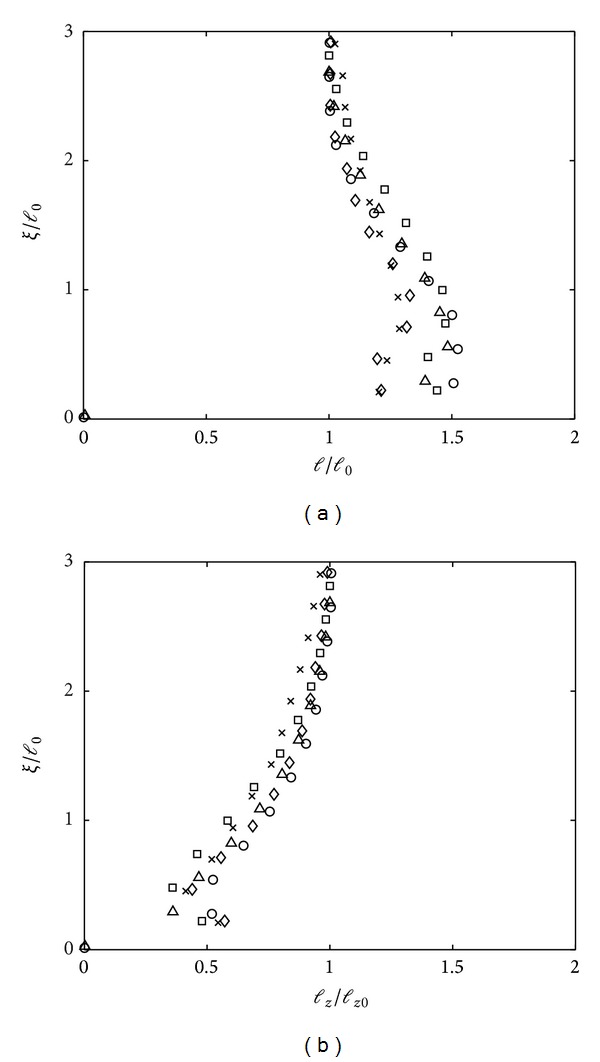
Plots showing measurements of integral length scales at near base for (a) horizontal *l* and (b) vertical *l*_*z*_ against dimensionless distance above the tank base *ξ*/*l*_0_. Refer to Figure 3 for legends of plots in (a) and (b), respectively.

Figure 5 shows the results of both horizontal and vertical integral length scales for flows *Re*_*l*_ > 117. The normalising factor *l*_*z*0_ denotes the value of *l*_*z*_ measured at depth *z* = 2.5*M* (similar approach when *l*_0_ was defined). The horizontal integral length scales *l* were seen to increase from *ξ*/*l*_0_ ≈ 1.5 and continued towards the base until *ξ*/*l*_0_ ≈ 0.5. The energetic eddies approach towards the boundary where the the eddies were stretched along the horizontal plane, increasing the diameter of the eddies. This is a rather similar behaviour observed for splat events, that is, when turbulent eddies or blobs of fluid moving towards the base are flattened and move parallel to the base surface, upon their impingement [3, 12]. The increasing size of *l* is seen to be dependent on the *Re*_*l*_, where flows with *Re*_*l*_ > 325 can have an increase up to *l*/*l*_0_ ≈ 1.5, whereas the maximum achieved for lower *Re*_*l*_ is around 1.3. The vertical integral length scales *l*_*z*_, as shown in Figure 5(b), were monotonically decreased in particular from *ξ*/*l*_0_ ≈ 1.5 towards the base. An increase of the *l*_*z*_ was observed at *ξ*/*l*_0_ ≈ 0.3, indicating an antisplat event, that is, the collision of two splats, resulting in an ejection of fluid (in the normal direction) from the base.

## 3. Results

Previous analysis shows that for flows *Re*_*l*_ ≥ 117, the turbulence generated using the described experimental setup changes according to the standard profile. Here, the experimental data obtained from flows generated at *Re*_*l*_ < 117 is discussed.

### 3.1. The Evolution of r.m.s Fluid Velocity

**Figure 6:**
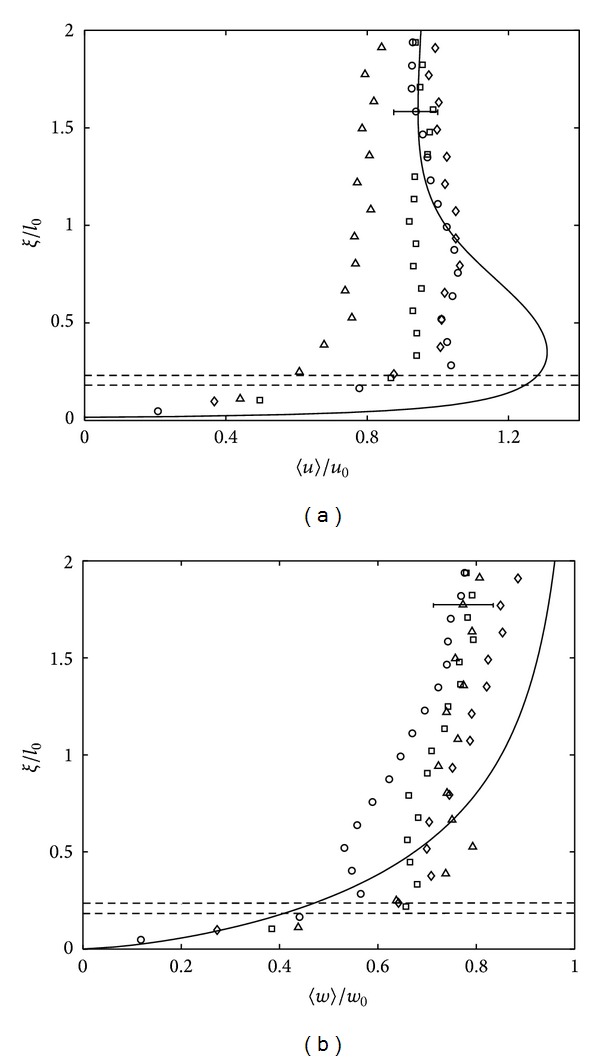
Plots showing measurements of (a) 〈*u*〉/*u*_0_ and (b) 〈*w*〉/*w*_0_ against dimensionless distance above the tank base *ξ*/*l*_0_. The data shown correspond to *Re*_*l*_ = 95 (∘), *Re*_*l*_ = 76 (□), *Re*_*l*_ = 74 (▵), and *Re*_*l*_ = 113 (*⋄*). To avoid saturation, a single error bar has been included in each plot which is representative of the variability observed in all of the data shown. The theoretical curves for both r.m.s horizontal and velocity components are included for comparison, shown here as solid lines.

The r.m.s velocity measurements of 〈*u*〉 and 〈*w*〉 within the blockage layer for flow conditions *Re*_*l*_ < 117 are plotted in Figure 6. For a consistent comparison, here the 〈*u*〉 and 〈*w*〉 were obtained at similar distances (as described in Section 2), that is, at *ξ*/*l*_0_ = 0–2 with the predicted lines also shown. The plots demonstrate contrasting profiles to the one obtained in flows with higher *Re*_*l*_ (as illustrated in Figure 3), suggesting appreciable changes in the turbulence structure within the blockage layer. The r.m.s horizontal velocity components experienced no amplification at 0 < *ξ*/*l*_0_ ≤ 0.5 and were consistently constant from 2⩾*ξ*/*l*_0_⩾0.3. This result is coherent with the plot of the normal velocity components (in Figure 6(b)), where they do not monotonically decrease and remain relatively constant from 2⩾*ξ*/*l*_0_⩾1. Thus no (significant) intercomponent energy transfer between the two velocity components occurred. Both r.m.s velocity profiles show a sharp decrease to zero (due to a viscous effect) from *ξ*/*l*_0_ ≈ 0.3, satisfying the no-slip condition at *ξ* = 0. The base does not possess any suppression effect for either horizontal or vertical fluid motions unlike the cases for high *Re*_*l*_.

The profiles shown in Figure 6 support the claim of Hunt and Graham [6], with regard to the importance of high *Re*_*l*_ for the occurrence of amplified 〈*u*_*p*_〉. It is believed that, at high *Re*_*l*_, the viscous effect is negligible within the blockage layer and the energetic eddies caused a local disruption on the viscous sublayer [20]. At low *Re*_*l*_, however, the dominant viscous stress dampens the energy and deviates the velocity profile from the standard curve. The viscous effect is evident as the thickness of the viscous sublayer (*δ*_*v*_) increases when *Re*_*l*_ decreases [21]. Using the relationship *δ*_*v*_ = 2*l*_0_ *Re*_*l*_^−1/2^, the relative values of *δ*_*v*_/*l*_0_ = for *Re*_*l*_ < 117 were calculated. The relative thickness of *δ*_*v*_ (for all data) spanned from 0 to 0.18 to 0.23*l*_0_, shown here in Figure 6 as (two) dashed lines. The thickness of the viscous sublayer attached to the base was (rather) a large fraction of the integral scale *l*_0_ and contributed to a sharp decrease of the tangential velocity components as the base is approached [6].

**Figure 7:**
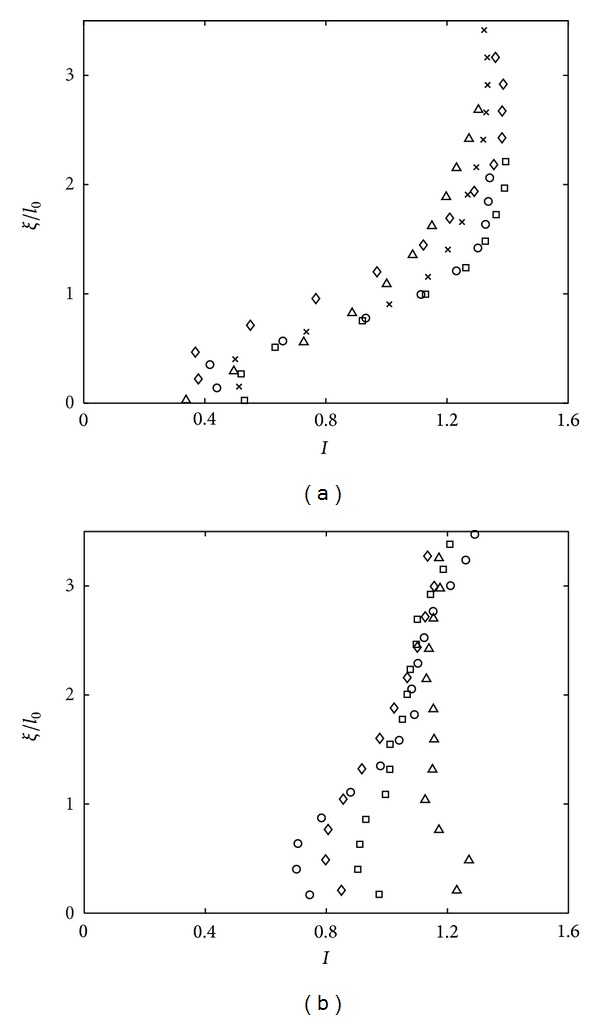
Plots showing measurements of isotropy for (a) *Re*_*l*_ ≥ 117 and (b) *Re*_*l*_ < 117 against dimensionless distance above the tank base *ξ*/*l*_0_. Refer to Figures 3 and 6 for legends of plots in (a) and (b), respectively.

The influence of the impermeability condition can also be traced through the evolution of isotropy *I* (defined as the ratio of spatially averaged 〈*u*〉 and 〈*w*〉). The plots of isotropy *I* = 〈*w*〉/〈*u*〉 for high *Re*_*l*_ and low *Re*_*l*_ at near-base were plotted in Figures 7(a) and 7(b), respectively, to give a direct comparison. The figures were plotted up to *ξ*/*l*_0_ = 3.5 to also include the quasi-isotropic homogeneous turbulence region.  Figure 7(a) shows that *I* is (consistently) constant between the range 1.2 < *I* < 1.4 from *ξ*/*l*_0_ = 2–3.5. Note that it is difficult (if not impossible) to achieve the ideal value *I* = 1 due to the normal forcing direction of the grid [22]. This is supported by the *I* obtained by Hopfinger and Toly [4], where *I* = *C*_2_/*C*_1_ = 1.08 (recall (1a) and (1b)). The isotropy plot (shown here in Figure 7) is not only to illustrate the isotropy behaviour within the flow but also to be used to identify the start of the blockage layer, that is, when *I* starts to decrease [3]. Data shows that, at *ξ*/*l*_0_ = 1.2, the previously constant *I* (at 1.2 < *ξ*/*l*_0_ < 3.5) experienced a decreasing value and monotonically decreased to zero at the base. Hence, the thickness of the blockage layer (using the experimental setup described) is ≈1.2*l*_0_ above the tank base.

For low *Re*_*l*_ (as shown in Figure 7(b)), however, *I* values were not constant and exhibit different and inconsistent profiles, in particular at near base. Isotropy remains relatively constant until *ξ*/*l*_0_ ≈ 1.2, before suppression or amplification of isotropy at *ξ*/*l*_0_ ≈ 0.3. At low *Re*_*l*_, the turbulence impacted on the base, which is perceived to be in a decaying state. In such conditions, the intercomponent energy transfer is crucially affected, where the intercomponent energy transfer near a flat (solid) surface depends crucially on the nature (steady vs decaying, homogeneous vs inhomogeneous) of the outer turbulence [21]. For such flow, using *I* to determine the blockage layer is not effective.

### 3.2. Integral Length Scales

**Figure 8:**
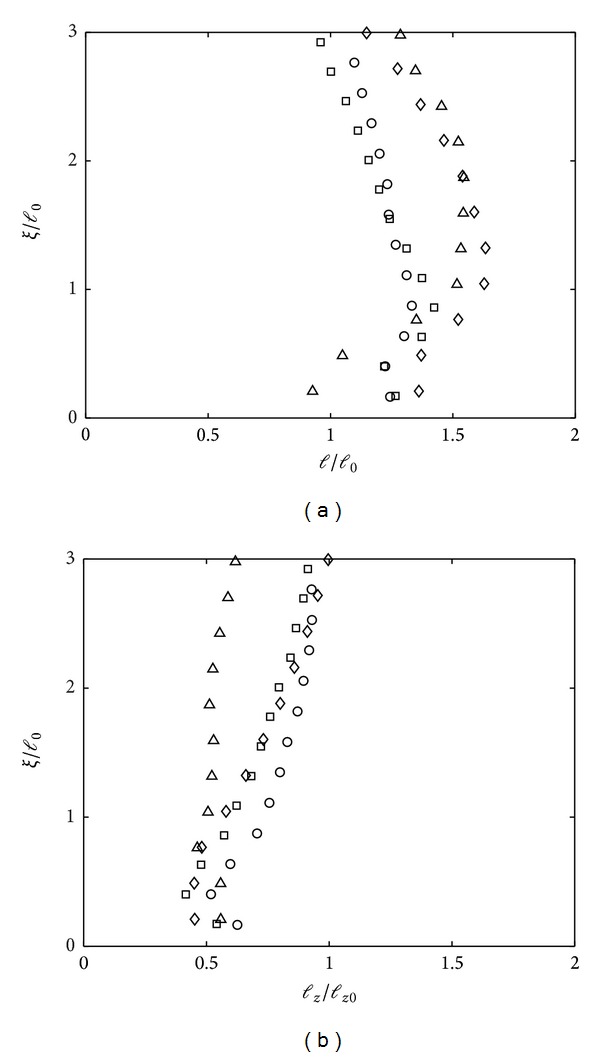
Plots of (a) horizontal (*l*) and vertical (*l*_*z*_) integral length scales against dimensionless distance above the tank base *ξ*/*l*_0_ for *Re*_*l*_ < 117. The data shown correspond to *Re*_*l*_ = 95 (∘), *Re*_*l*_ = 76 (□), *Re*_*l*_ = 74 (▵), and *Re*_*l*_ = 113 (*⋄*).

Figure 8 shows the measurements of both horizontal *l* and vertical *l*_*z*_ integral length scales, made dimensionless with their respective values at *z* = 2.5*M*. In the case of a solid boundary with *w* → 0 at the wall and at a distance *ξ* < *l*_0_, the eddies are flattened due to the impingement on the base. It can be said here that although it is not distinctive as the trends shown in Figure 5, the splat-antisplat events occur, even at low *Re*_*l*_. An increase of *l* towards the base was evident for some flows (for example, *Re*_*l*_ = 113 (⋄) shown in Figure 8) and accompanied by a consistent decreasing *l*_*z*_ until *ξ*/*l*_0_ ≈ 0.3 before experiencing a slight increase at near base.

Particular attention is paid for the lowest flow *Re*_*l*_ = 74 (symbol ▵), where at near base, *l*_*z*_ relatively experienced no changes within the blockage layer, in contrast with the horizontal integral length scales where *l* was increased up to *l*/*l*_0_ ≈ 1.6. The profiles indicate that the splat events occur due to low *Re*_*l*_, where the pressure strain term decreases and subsequent increase of the effect from viscosity reduces the possibility of antisplat events [12]. Changes of the horizontal and vertical integral length scales within the blockage layer at *Re*_*l*_ > 74 suggest that the presence of base induced splat-antisplat events. The occurrence of the antisplat event is dependent on the *Re*_*l*_, where at low *Re*_*l*_ (for example, here at *Re*_*l*_ = 74) the incoming turbulence at near base was in a decaying state, such that the energy contained within the eddies is weak and the strong viscous effects near the base dampen the energy.

### 3.3. Spectra

The fluid velocity measurement using PIV allows the analysis of simultaneous time series velocity components at various distances. Measurement of one-dimensional energy spectra at different distances above the base, at a distances of *ξ* = 0.05, 0.5, 1, and 2 cm, respectively, was performed. The energy spectra were calculated from the time series of horizontal and vertical velocity components, for each distance. Let *f*(*t*_*n*_) be an input signal (a sequence) of either horizontal or vertical velocity time series and let *X*(*f*) be a frequency spectrum; the result of the discrete Fourier transformation of a signal *f*(*t*_*n*_) is expressed as

(5) $$\begin{array}{c} X\left(f\right)=\overset{N-1}{\underset{n=0}{\sum }}f\left(t_{n}\right)e^{-(2\pi i/N)nf}, \end{array}$$

where *n* = 0,1,…, *N* − 1, *i* is imaginary unit and *e* ^−2*πi*/*N*^ is a primitive *N*th root of unity.

**Figure 9:**
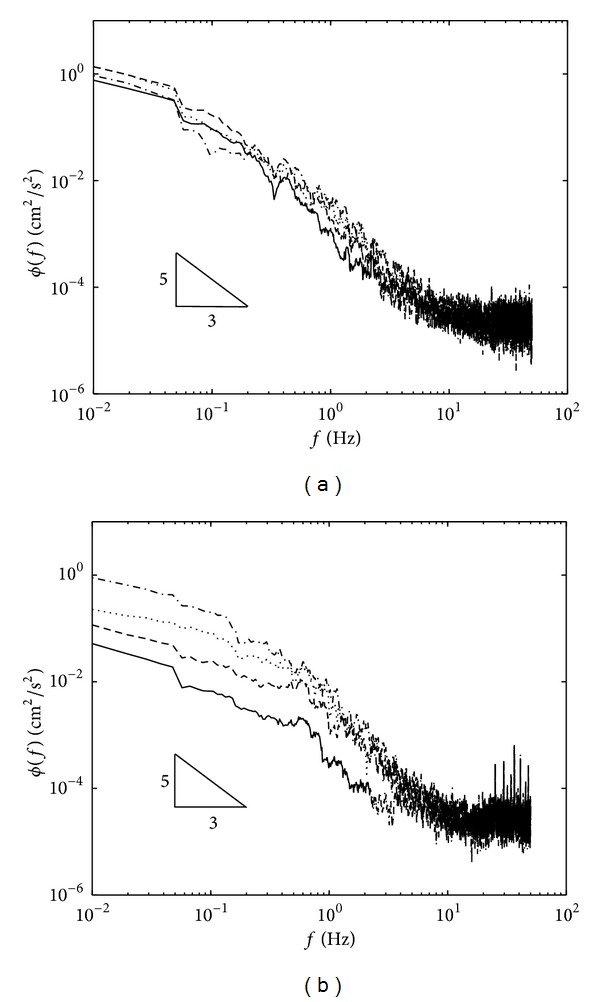
Plots showing the one dimensional frequency spectrum for (a) *ϕ*_*u*_(*f*) and (b) *ϕ*_*w*_(*f*) for a typical high *Re*_*l*_ flow. The experiments shown correspond to *Re*_*l*_ = 474 at *ξ*/*l*_0_ = 0.032 (solid), 0.317 (dashed), 0.634 (dotted), and 1.27 (dash-dotted). The triangle-solid shows the slope *f* ^−5/3^.

**Figure 10:**
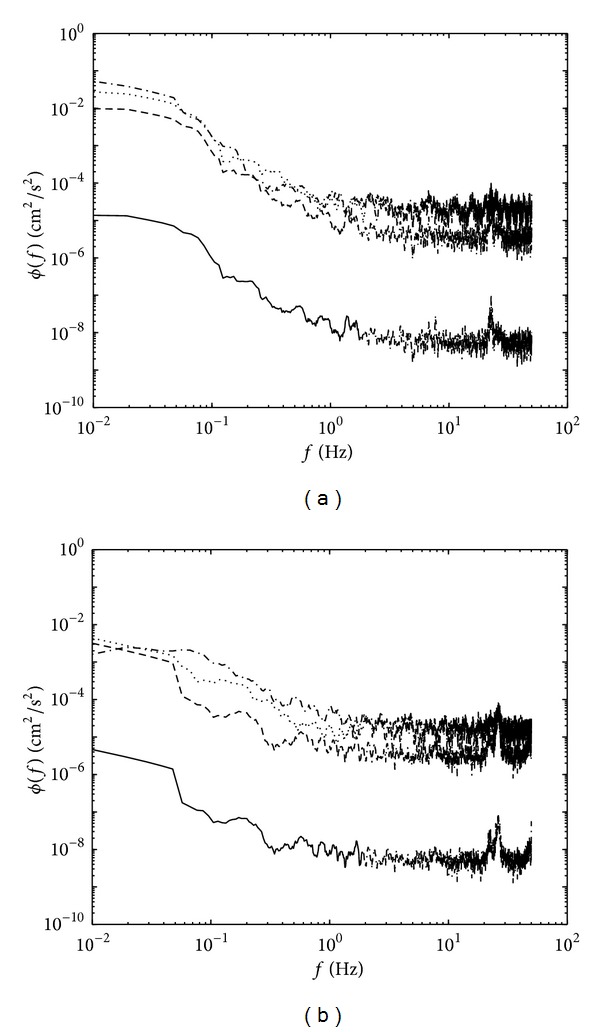
Plots showing the one dimensional frequency spectrum of (a) *ϕ*_*u*_(*f*) and (b) *ϕ*_*w*_(*f*) for a typical low *Re*_*l*_ flow. The experiments shown correspond to *Re*_*l*_ = 113 at *ξ*/*l*_0_ = 0.032 (solid), 0.317 (dashed), 0.634 (dotted), and 1.27 (dash-dotted).

By performing a fast fourier transformation to compute the relations in (5), the energy spectral density is calculated as

(6) $$\begin{array}{c} \phi \left(f\right)=\frac{X\left(f\right)X^{*}\left(f\right)}{2\pi }, \end{array}$$

where *X*(*f*) and X*(*f*) are the real and imaginary components, respectively. The horizontal and vertical energy spectra which are denoted by *ϕ*_*u*_(*f*) and *ϕ*_*w*_(*f*), respectively, were obtained using (5) and (6), shown in Figures 9 and 10 for flows of *Re*_*l*_ ≥ 117 and *Re*_*l*_ < 117, respectively.

The plot of high *Re*_*l*_ ≥ 117 will be discussed first, where the one-dimensional spectra for velocity components, taken at near base up to the homogeneous region (i.e., at *ξ*/*l*_0_ = 0.032, 0.317, 0.634, and 1.27), were plotted together. In Figure 9 the frequency spectrum, which represents a homogeneous region, is plotted as dashed-dotted lines for both *ϕ*_*u*_(*f*) and *ϕ*_*w*_(*f*). As the Reynolds number *Re*_*l*_ here is relatively small compared to the Reynolds number in naturally occurring turbulent flow, the *f* ^−5/3^ slope (showing inertial subrange) is not guaranteed. Restricting the Reynolds number restricts the frequency range; that is, the range may not be wide enough to exhibit an inertial subrange. However, the data in Figure 9 suggests that the inertial subrange exists within the frequency range of 0.078 ≤ *f* ≤ 1 for the horizontal component and at a frequency range of 0.4 ≤ *f* ≤ 1.5 for the vertical component. As the base is approached, the inertial subrange region loses its characteristics and very near to the base (at *ξ*/*l*_0_ = 0.032) an inertial subrange barely exists.

The plot shows that as the base was approached, the horizontal components received more kinetic energy and were seen to amplify at *ξ*/*l*_0_ = 0.317. Very near to the base *ξ*/*l*_0_ = 0.032, the energy decreases satisfying the no slip boundary condition. The amplification of *ϕ*_*u*_(*f*) is due to the transferred energy from the vertical components, where *ϕ*_*w*_(*f*) must decrease to satisfy the kinematic boundary condition, as supported in the analysis shown in Figure 9(b). The presence of tank base has no effect on the high frequency end of the energy spectrum, implying that the net transfer of turbulent kinetic energy from the vertical to the horizontal components of velocity is confined to small frequencies or large eddy scales. Note that similar profiles were obtained for all data and Figure 9 is a representative profile for all data.

The energy spectrum for *Re*_*l*_ < 117, shown here in Figure 10, evidently demonstrates low energy for both *ϕ*_*u*_(*f*) and *ϕ*_*w*_(*f*). Note that the distance *ξ* taken was consistent with the distance shown in Figure 9 (i.e., at 0.05, 0.5, 1, and 2 cm) above the tank base. The energy for horizontal and vertical components steadily decreased as they approached the solid surface, notably at the low-frequency end. No amplification of kinetic energy in the horizontal direction was observed. At low *Re*_*l*_, the flow is believed to have reached the state of decaying turbulence as it approached the tank base. At this state, the viscous effects near the base inhibit the amplification of the horizontal component [12]. The *ϕ*_*u*_(*f*) were found to decrease as *ξ* → 0, where the kinetic energy was progressively dampened by the viscous forces for both large and small scale eddies. Similarly, the plot of *ϕ*_*w*_(*f*) (as shown in Figure 10(b)) evidently shows a comparable profile with *ϕ*_*u*_(*f*). The kinetic energy for both low and high frequency ends steadily decreased towards the tank base, suggesting that the range of sizes of active eddies contributing to the energy spectrum decreased. At low flows of *Re*_*l*_, the energy transfer from the large-scale eddies to the viscous dissipating (small-scale) eddies is much faster, as the external energy (from the movement of oscillating-grid) at high frequency end is low. Both energy spectrums eventually approached zero in a very thin viscous layer where measurement was made very close to the tank base (i.e., *ξ*/*l*_0_ = 0.032), shown as solid lines in Figure 10, showing a low energy of ≈10^−5^. Note that the noise peak at ≈23 Hz in Figures 10(a) and 10(b) is speculated to be caused by mechanical vibration, probably due to the grid oscillation.

## 4. Conclusions

The experimental work presented here described the characteristics of experimentally oscillating-grid generated turbulence at impermeable surface, for which the base of an acrylic tank was used. The motivation for the study was to find the lower limit of *Re*_*l*_, where the turbulence profile at near base follows the rapid distortion theory based on the calculation of Hunt and Graham [6]. Data was obtained from a series of experiments with the turbulent Reynolds number ranging between 74 ⩽ *Re*_*l*_ ⩽ 570. Although there are apparent discrepancies between the assumptions of the theory and the experimental conditions, there was clear qualitative agreement between our data trend and that predicted by Hunt and Graham [6] for flows of *Re*_*l*_⩾117. The r.m.s horizontal velocity components amplified at *ξ*/*l*_0_ ≈ 0.3, where the energy is transferred from the decreasing vertical velocity components within the blockage layer. The changes of velocity components mimic the splat-antisplat events, where the eddies flatten, increase in diameter, and eject back the outer fluid. Additionally, the occurrence of splat events was evidenced by a notable increment of horizontal integral length scales and associated monotonous decrease of vertical integral length scales within the blockage layer. Subsequent antisplat events were observed where the vertical length scales *l*_*z*_ were seen to increase at *ξ*/*l*_0_ ≈ 0.3.

For flows *Re*_*l*_ < 117, however, the r.m.s horizontal velocity components experienced no amplification. Both 〈*u*〉 and 〈*w*〉 were relatively constant towards the tank base and sharply decreased to zero at *ξ* ≈ 0.3*l*_0_ due to the viscous effect. The increasing and simultaneously decreasing of horizontal and vertical integral lengthscales, respectively, suggest that, even at low *Re*_*l*_ > 74, the splat-antisplat events occurred. However, at the lowest *Re*_*l*_ = 74 (available in this study), only splat event was evident. The strong viscous effect at near base dampens the available tangential energy to drive the antisplat events.

The incoming flow *Re*_*l*_ < 117 impinging on the tank base showed the characteristics of anisotropic and alters the turbulence profile at near tank base. For flows of *Re*_*l*_ < 117, the tank base poses quite an impact on both large and small-scale eddies, where their kinetic energy monotonously decreases when approaching the base. A direct quantitative comparison of the velocity components for flows *Re*_*l*_ < 117 with the Hunt and Graham [6] model shows a departure from the theoretical prediction. For low flows *Re*_*l*_ < 117, the turbulence at near base was damped, largely contributed by the significant presence of the viscous sublayer.

When using an oscillating grid to investigate the sole effect of turbulence on geophysical phenomena, the lower limit of *Re*_*l*_ is within the range 114 ⩽ *Re*_*l*_ ⩽ 116. However, we recognised that the precise range may differ with each experimental setup. This research narrowed down the previous possible range *Re*_*l*_ limit (i.e., 40 < *Re*_*l*_ < 150) that does not follow the theoretical profiles of Hunt and Graham [6] to a much smaller range of 114 ⩽ *Re*_*l*_ ⩽ 116, where this can act as definitive limit. That is, for any experiments conducted below this value, a different physical interpretation of the turbulence at near base is acquired.

## Figures and Tables

**Table 1:** A summary of the *u*_0_, *w*_0_, and *l*_0_ for each ow studied.

*Re*_*l*_	*u*_0_ (cm/s)	*w*_0_ (cm/s)	*l*_0_ (cm)
Flows with high *Re*_*l*_			
570	1.606	2.154	1.78
474	1.502	2.094	1.58
325	1.336	1.742	1.22
209	0.604	0.871	1.73
117	0.570	0.814	1.35
Flows with low *Re*_*l*_			
113	0.482	0.556	1.18
95	0.341	0.449	1.40
76	0.297	0.378	1.28
74	0.302	0.361	1.22
